# An archaeal virus capable of hydrolyzing the surface glycan of the host cell

**DOI:** 10.1002/mlf2.70008

**Published:** 2025-04-03

**Authors:** Wanjuan Yuan, Caixia Pei, Junkai Huang, Hongyu Chen, Juanying Fan, Cheng Jin, Li Huang

**Affiliations:** ^1^ Key Laboratory of Microbial Pathogenesis and Interventions of Fujian Province University, the Key Laboratory of Innate Immune Biology of Fujian Province, Biomedical Research Center of South China, College of Life Sciences Fujian Normal University Fuzhou China; ^2^ State Key Laboratory of Microbial Resources, Institute of Microbiology Chinese Academy of Sciences Beijing China; ^3^ State Key Laboratory of Mycology, Institute of Microbiology Chinese Academy of Sciences Beijing China; ^4^ University of Chinese Academy of Sciences Beijing China; ^5^ Southern Marine Science and Engineering Guangdong Laboratory (Guangzhou) Guangzhou China; ^6^ Center for Geomicrobiology and Biogeochemistry Research, State Key Laboratory of Biogeology and Environmental Geology China University of Geosciences Beijing China

## Abstract

Spindle‐shaped viruses exclusively infect archaea. Fuselloviruses represent a large group of spindle‐shaped viruses and infect hyperthermophilic archaea of the order *Sulfolobales*. Although the first fusellovirus was identified nearly 40 years ago, the mechanism of host infection by these viruses remains poorly understood. Here, we show that SSV19, a fusellovirus isolated from a hot spring in the Philippines, is capable of hydrolyzing the host cell surface glycan identified as a heptasaccharide chain of QuiS_1_Hex_4_HexNAc_2_. Our findings provide significant insights into the molecular strategy of host recognition and, possibly, entry by an archaeal virus.

Host recognition and entry represent crucial steps in the lifecycle of a virus. However, the current knowledge of these initial steps in archaeal viruses, which are often found in extreme habitats and exhibit extraordinary morphologies, is fragmentary and limited^1^, ^2^. *Saccharolobus islandicus* rod‐shaped virus 2 (SIRV2) was shown to bind specifically to the tips of pilus‐like filaments of the host cell before entry^3^. *Sulfolobus* turreted icosahedral virus (STIV) also recognizes pilus‐like structures with its surface turret proteins^4^. *Haloarchaeal* pleomorphic virus 6 (HRPV6) enters the host cell through membrane fusion mediated by a spike protein^5^. Spindle‐shaped *Sulfolobus* monocaudavirus 1 (SMV1) was proposed to employ a possible fusion entry mechanism^6^. In general, however, molecular mechanisms underlying the early steps of interaction between archaeal viruses and their hosts remain largely unclear.

We have been investigating early steps in the interaction between *Sulfolobus* spindle‐shaped virus 19 (SSV19), a member of the genus *Betafusellovirus* of the family *Fuselloviridae*, and the host *Sulfolobus* sp. E11‐6. Spindle‐shaped viruses, which infect archaea exclusively and exist in acidic hot springs, hypersaline and marine environments, are grouped into four families, i.e., *Bicaudaviridae*, *Thaspiviridae*, *Halspiviridae*, and *Fuselloviridae* ^7^. SSV19 and *Sulfolobus* sp. E11‐6 were isolated from a sediment sample from a hot spring in the Philippines, and characterized^8^. Recently, we obtained a cryo‐EM structure of SSV19, and showed that the capsid shell of the virion is formed by seven left‐handed helical strands, and is attached to a sevenfold symmetrical tail consisting of nozzle protein C131, adaptor protein B210, and tailspike protein VP4^9^. Intriguingly, VP4 contains a putative endo‐mannanase domain, which shares structural similarity with the *Bacteroides thetaiotaomicro* endo‐mannanase shown to be active on yeast α‐mannan, a densely branched *N*‐linked glycan on the surface of yeast cell walls^10^.

To learn if the mannanase domain of VP4 serves a potential role in the SSV19‐host interaction, we first sought to determine the structure of glycans on the surface of the host cell. The *N*‐glycans were released from the host cells using the non‐reductive *β*‐elimination method and hydrolyzed with trifluoroacetic acid (TFA)^11^. The hydrolysate contained glucosamine (GlcNAc), glucose (Glc), and mannose (Man), as revealed by high‐performance anion‐exchange chromatography with pulsed amperometric detection (HPAEC‐PAD) (Figure 1A).

**Figure 1:**
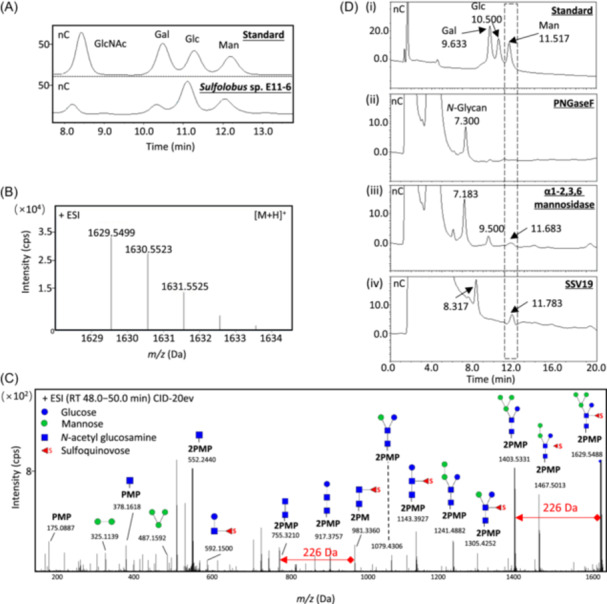
Analysis of *N*‐glycans and their hydrolysis by SSV19. (A) Profiles of a mixture of monosaccharide standards containing *N*‐acetylglucosamine (GlcNAc), galactose (Gal), glucose (Glc) and mannose (Man) (top), and the trifluoroacetic acid (TFA) hydrolysate of *N*‐glycan (bottom) revealed by high‐performance anion‐exchange chromatography with pulsed amperometric detection (HPAEC‐PAD). (B) Mass spectrum of 1‐phenyl‐3‐methyl‐5‐pyrazolone (PMP) labeled *N*‐glycans from *Sulfolobus* sp. E11‐6. (C) MS^2^ spectra of *N*‐glycan (*m/z*, 1629.55). The 226‐Da molecule (red arrow) derived from peaks at [M + H]^+^ *m/z* 1629.55 and *m/z* 1403.53 is presumably sulfoquinovose (QuiS). The fragment peaks at [M + H]^+^ *m/z* 755.32 and [M + H]^+^ *m/z* 981.34 suggest that QuiS is attached to *N*‐acetylhexosamine. (D) HPAEC‐PAD analysis of the host cell *N*‐glycan incubated with mannosidases or SSV19. (i) A standard mixture of Gal, Glc, and Man at 50 μg/ml each. (ii) *N*‐glycans released from *Sulfolobus* sp. E11‐6 by PNGaseF. (iii and iv) Product analysis of the *N*‐glycans from *Sulfolobus* sp. E11‐6 treated with α1‐2,3,6 mannosidase (iii) or SSV19 virions (iv).

The *N*‐glycans were also released from the membrane proteins of *Sulfolobus* sp. E11‐6 under alkaline conditions using the one‐pot method^12^. The reducing ends of the glycans were labeled with two molecules of 1‐phenyl‐3‐methyl‐5‐pyrazolone (PMP) and analyzed by liquid chromatography‐tandem mass spectrometry (LC‐MS^2^). As shown in Figure 1B, the *N*‐glycan chain labeled with the PMP formed a peak at the [M + H]^+^ *m/z* of 1629.55 in the positive ion mode. Based on the MS^2^ spectra of peak [M + H]^+^ *m/z* 1629.55, we inferred that the molecular mass of *N*‐glycan is 1289 Da (Figure 1C). The MS^2^ spectra suggest that the *N*‐glycan from the host cell comprises two *N*‐acetylhexosamines, four hexoses, and a 226‐Da sugar residue. This 226‐Da molecule derived from peaks at [M + H]^+^ *m/z* 1403.53 and *m/z* 1629.55 is presumably sulfoquinovose (QuiS), as found in glycan chains from other *Sulfolobus* species^13^, ^14^, ^15^. The fragment peaks at [M + H]^+^ *m/z* 755.32 and [M + H]^+^ *m/z* 981.34 suggest that QuiS is attached to *N*‐acetylhexosamine rather than hexose.

Based on these observations as well as the monosaccharide composition analysis of the glycan, we conclude that the *N*‐glycan of *Sulfolobus* sp. E11‐6 is the heptasaccharide QuiS_1_Hex_4_HexNAc_2_. Several glycan structures have been reported for different *Sulfolobus* strains^13^, ^14^, ^15^. Our results suggest that the *N*‐glycan of *Sulfolobus* sp. E11‐6 is similar to that reported in *Sulfolobus tokodaii* (Figure 1C)^13^.

We subsequently set out to test if VP4 from SSV19 is able to hydrolyze the host cell glycan, as suggested by the structural similarity between the tailspike protein and the bacterial endo‐mannanase. We first treated the host cells with PNGaseF, an enzyme capable of cleaving *N*‐linked oligosaccharides between the innermost GlcNAc and asparagine residues from glycoproteins^15^. As expected, only a single peak was detected by HPAEC‐PAD, indicating that the host *N*‐glycan was specifically released (Figure 1D‐ii). We then tried to prepare recombinant VP4 in *Escherichia coli* and *S. islandicus*. However, our repeated attempts were unsuccessful presumably due to the hydrophobicity of this large protein (137 kDa). This prompted us to test directly the ability of the SSV19 virion to hydrolyze the host cell glycan. Significantly, a mannose peak was detected as the cleavage product following the incubation of the SSV19 particles with the host glycan at 75°C overnight, and the *N*‐glycan peak (7.1–7.3 min) was shifted to 8.3 min, suggesting the host *N*‐glycans were completely converted into smaller oligosaccharides and mannose (Figure 1D‐iv). As a positive control, incubation of the host glycan with a mixture of α‐mannosidases also yielded mannose and a smaller oligosaccharide (9.5 min). However, it appears that α‐mannosidases were not as efficient as the virus particles in the cleavage of the host *N*‐glycans (Figure 1D‐iii). Therefore, we conclude that the SSV19 virion is able to recognize and degrade the glycan on the host cell surface. Since VP4 is the only structural protein of SSV19 that has been predicted to possess hydrolytic activity toward carbohydrates, we speculate that the observed host glycan cleavage is attributed to the activity of the tailspike protein of the virus. The structural proteins of some viruses have been shown or suggested to be derived from host enzymes, and some of these structural proteins may have lost enzymatic activity as the viruses evolve^16^, ^17^. To the best of our knowledge, this is the first experimental demonstration of the hydrolytic activity of an archaeal virion on the host glycan. Bacteriophage φ29 tailspikes have been shown to hydrolyze peptidoglycan, facilitating the penetration of the tail into the host cell wall during the process of phage entry^18^. Conceivably, the hydrolysis of host surface glycan by the SSV19 virion may play a similar role in SSV19 infection.

A search in IMG/VR v4, an expanded database of uncultivated virus genomes within a framework of extensive metadata^19^, retrieved 73 VP4 homologues (3 iterations, *e*‐value <10) (Table S1). Sequences with query coverage greater than 50% were selected for further analysis. The homologous proteins are primarily from hot springs in Yellowstone National Park, USA; Hveragerdi, Iceland; Naghaso, Philippines; and Tengchong, China (Figure S1A). As revealed by sequence alignment, five key residues in the catalytic domain of the bacterial endo‐mannanase are conserved in eleven selected VP4 homologues^9^ (Figure S1B). The structure of a VP4 homologue (Ga0187310_140793) was predicted with AlphaFold3^20^. Structural comparison shows that it indeed contains a domain similar to the core domain of SSV19 VP4 (Figure S1C). Therefore, virus‐host interaction involving a VP4 homologue may occur widely in viruses, most likely archaeal viruses, thriving in thermoacidic habitats. Taken together, our data shed significant light on the mechanism underlying the host recognition and, possibly, entry by an archaeal virus.

## AUTHOR CONTRIBUTIONS

**Wanjuan Yuan**: Data curation; methodology; writing—original draft; writing—review and editing. **Caixia Pei**: Data curation; formal analysis; methodology; visualization; writing—review and editing. **Junkai Huang**: Data curation; software; visualization. **Hongyu Chen**: Methodology; software; visualization. **Juanying Fan**: Methodology. **Cheng Jin**: Conceptualization; project administration; supervision; writing—review and editing. **Li Huang**: Conceptualization; funding acquisition; project administration; resources; supervision; writing—review and editing.

## ETHICS STATEMENT

This study did not involve any human participants or animal subjects.

## CONFLICT OF INTERESTS

The authors declare no conflict of interests.

## Supporting information

Supporting information.

**Figure S1.** Phylogenetic analysis of VP4. (A) Phylogenetic trees of SSV19 VP4 and its homologues. Proteins homologous to VP4 were collected using PSI‐BLASTP. Details of the analysis are described in the Materials and Methods section. SSV19 VP4 is marked with a red dot. The sampling location, pH, and temperature of VP4 and its homologues are shown in different colors on the right. (B) Sequence alignment of SSV19 VP4 and its homologues. Five key residues (i.e., Phe66, Asp69, Asp70, Tyr640, and Trp1033) in the VP4 core domain are marked with a star. Eleven homologues that have the same five residues with VP4 are marked with a red dot. (C) Comparison of the structural domains of Ga0187310_140793 (cyan) and SSV19 VP4 (brown). The key residues are shown.

Supporting information.

## Data Availability

The authors confirm that the data supporting the findings of this study are available within the article and its Supporting Information Materials.
