# Supplementary material for: An archaeal virus capable of hydrolyzing the surface glycan of the host cell
Source: mLife. 2025 Apr 3;4(2):219–22. doi: 10.1002/mlf2.70008 (PMC12042106; doi:10.1002/mlf2.70008)
Supplement: Supplementary file 1 — Supporting information. [file MLF2-4-219-s003.docx]

**Supporting Information**

**An archaeal virus capable of hydrolyzing the surface glycan of the host cell**

Wanjuan Yuan^1,2,#^, Caixia Pei^3,6,#^, Junkai Huang^4^, Hongyu Chen^5^, Juanying Fan^3^, Cheng Jin^3,^^6,^*, and Li Huang^2,4,6,^*

^1^Key Laboratory of Microbial Pathogenesis and Interventions of Fujian Province University, the Key Laboratory of Innate Immune Biology of Fujian Province, Biomedical Research Center of South China, College of Life Sciences, Fujian Normal University, Fuzhou, 350117, China.

^2^State Key Laboratory of Microbial Resources, Institute of Microbiology, Chinese Academy of Sciences, Beijing, 100101, China.

^3^State Key Laboratory of Mycology, Institute of Microbiology, Chinese Academy of Sciences, Beijing, 100101, China.

^4^Southern Marine Science and Engineering Guangdong Laboratory (Guangzhou), Guangzhou, 511458, China.

^5^Center for Geomicrobiology and Biogeochemistry Research, State Key Laboratory of Biogeology and Environmental Geology, China University of Geosciences, Beijing 100083, China.

^6^University of Chinese Academy of Sciences, Beijing, 100049, China.

*Correspondence to: huang_li@gmlab.ac.cn and jinc@im.ac.cn

^#^These authors contributed equally to this work.

**Materials and methods**

**Purification of SSV19 virions**

*Sulfolobus* sp. E11-6 infected with SSV19 was grown with shaking at 75°C in PSCVY medium to an OD_600_ of ~ 1.0, and the culture was centrifuged at 5000 × *g* for 20 min at 4°C^1^. The supernatant was filtered through a 0.45-μm filter and centrifuged at 120,000 × *g* for 1 h at 4℃. The pellet was resuspended in Zillig’s basal salts, and subjected to cesium chloride density gradient centrifugation with a final CsCl concentration of 0.45 g/mL at 200,000 × *g* for 24 h at 4°C. The purified SSV19 particles were washed with Zillig’s basal salts by ultrafiltration through a 30-kDa centrifugal filter unit (Merck Millipore).

**Extraction of host membrane proteins**

*Sulfolobus* sp. E11-6 was cultured in PSCVY medium to an OD_600_ of ~ 1.0, and the culture (200 mL) was centrifuged at 5000 × *g* for 10 min at 25°C. The pellet was resuspended in 20 mM Tris-HCl, pH 6.5, and the cells were sonicated (5 s on, 7 s off, 35% power, 35 min). The supernatant was collected after centrifugation at 7,500 × *g* for 20 min at 4°C, and subjected to ultracentrifugation at 200,000 × *g* for 60 min at 4°C. The pellet was resuspended in 1% Triton X-100 (1 mL), incubated at 37°C with shaking at 150 rpm for 30 min, and then centrifuged again at 12,000 × *g* for 20 min at 4°C. The supernatant was dialyzed overnight against 20 mM Tris-HCl, pH 6.5, 0.05% Triton X-100, and then lyophilized for storage. Protein concentration is measured using a Bradford assay kit with bovine serum albumin (BSA) as the standard.

**Analysis of *N*-glycans labeled with PMP**

In order to analyze the structure of *N*-glycan by HPLC-MS/MS, glycans from host membrane proteins (1 mg) were released and labeled with PMP under alkaline conditions using a one-pot method as described previously^2^. The labeled *N*-glycans were separated on an Agilent HC-C18 column (250 × 4.6 mm, 5 μm). The primary mass spectrometry signals within the 100-2,000 *m/z* range in positive ion mode were collected, and subjected to MS/MS using CID with collision energies ranging from 10 to 30 v. Agilent MassHunter Workstation Software-Qualitative Analysis was employed for the analysis and identification of glycans.

**Analysis of monosaccharide components of glycan**

To analyze its monosaccharide composition, the glycan was released from the host membrane proteins by the non-reductive *β*-elimination method as described previously^3^. Purified glycan was dissolved in 200 μL of 2 M trifluoroacetic acid (TFA) and hydrolyzed to monosaccharide for 2 h at 115°C and then blown-dry. The monosaccharides of the glycan and standards were analyzed by HPAEC-PAD (high performance anion exchange chromatography-pulsed amperometric detector) (Dionex). Standards included *N*-acetylglucosamine, glucose, galactose, and mannose. Separation was performed using a CarboPac™ PA10 IC column (250 × 4 mm) under the following conditions: 18 mM NaOH at a flow rate of 1.0 mL/min.

**Hydrolytic activity assays**

Since the *N*-glycan was found to contain N-acetylglucosamine (GlcNAc), a more efficient enzymatic method, involving the use of PNGaseF, was employed to release the *N*-glycan. The cutting site of PNGaseF is the amide bond between GlcNAc and the glycoprotein^4^*. N*-glycans were specifically released from the host membrane proteins with PNGaseF (NEB) following incubation at 37^o^C for 48 h. Upon the addition of two volumes of ethanol, the proteins were precipitated. The supernatant was concentrated to complete dryness using a SpeedVac concentrator. Finally, the dried glycans were used as a substrate for subsequent experiments. The N-glycan dissolved in deionized water as a blank control and analyzed with a CarboPac™ PA10 IC column (250 × 4 mm).

Hydrolysis of *N*-glycans was carried out using α1-6 mannosidase and α1-2,3 mannosidase (NEB) . The reaction was incubated at 37°C for 12 h in a buffer containing 5 mM CaCl_2_ and 50 mM sodium acetate at pH 5.5. After reaction, two volumes of ethanol were added to inactivate the enzyme, and the precipitated proteins were then removed by centrifugation. The supernatant was concentrated and analyzed with a CarboPac™ PA10 IC column (250 × 4 mm).

The hydrolytic activity of the SSV19 particles (100 μL; ~ 1.5 x 10^13^ copies/μL) on the *N*-glycans from the host cell membrane were performed at 75°C for overnight in Zillig’s basic salt^1^. After reaction, the sample was centrifuged in a 3-kDa ultrafiltration tube at 8,000 x *g* for 20 min at 4^o^C to remove the virus particles. The filtrate was treated with two volumes of ethanol and centrifuged. The supernatant was dried, and the hydrolysate and standards were analyzed as above.

**Phylogenetic analysis**

A PSI-BLAST^5^ search was performed to identify proteins sharing homologous amino acid sequences with the SSV19 VP4 protein in the IMG VR v4 database^6^. A total of 73 sequences were retrieved with an E-value threshold of <10, using three iterations. Sequences with query coverage greater than 50% were selected for further analysis. A total of 21 sequences were chosen for phylogenetic analysis. Multiple sequence alignment was performed using L-INS-I^7^, and a phylogenetic tree was constructed using IQ-TREE2^8-10^ with the maximum likelihood method. The parameters applied included -m MFP, -madd LG4M, LG4X, -mrate E, I, G, I+G, R, -cmax 8, and -bb 1000. Based on the Akaike Information Criterion (AIC), corrected AIC (AICc), and Bayesian Information Criterion (BIC), the LG+F+R3 model was selected as the best-fitting model from a set of 398 candidates.

Sequence alignment of SSV19-VP4 and its homologues was performed and the results were displayed by DNAMAN software.

**AlphaFold3 prediction**

The structures of VP4 homologues were predicted using AF3^11^ via AlphaFold Server (<https://alphafoldserver.com/>). The best model (ranked_0.pdb) was obtained according to the average pLDDT score.

**Reference**

1 Han Z, Yuan W, Xiao H, Wang L, Zhang J, Peng Y, et al. Structural insights into a spindle- shaped archaeal virus with a sevenfold symmetrical tail. *Proc Natl Acad Sci USA*. 2022;119:e2119439119.

2 Pei C, Lu H, Ma J, Eichler J, Guan Z, Gao L, et al. AepG is a glucuronosyltransferase involved in acidic exopolysaccharide synthesis and contributes to environmental adaptation of Haloarcula hispanica. *J Biol Chem*. 2023;299:102911.

3 Lu H, Pei C, Zhou H, Lu Y, He Y, Li Y, et al. Agl22 and Agl23 are involved in the synthesis and utilization of the lipid-linked intermediates in the glycosylation pathways of the halophilic archaeaon Haloarcula hispanica. *Mol Microbiol*. 2020;114:762-74.

4 Palmieri G, Balestrieri M, Peter-Katalinic J, Pohlentz G, Rossi M, Fiume I, et al. Surface-exposed glycoproteins of hyperthermophilic Sulfolobus solfataricus P2 show a common N-glycosylation profile. *J Proteome Res*. 2013;12:2779-90.

5 Altschul SF, Madden TL, Schäffer AA, Zhang J, Zhang Z, Miller W, et al. Gapped BLAST and PSI-BLAST: a new generation of protein database search programs. *Nucleic Acids Res*. 1997;25:3389-402.

6 Camargo AP, Nayfach S, Chen I-MA, Palaniappan K, Ratner A, Chu K, et al. IMG/VR v4: an expanded database of uncultivated virus genomes within a framework of extensive functional, taxonomic, and ecological metadata. *Nucleic Acids Res*. 2022;51:D733-D43.

7 Katoh K, Standley DM. MAFFT Multiple Sequence Alignment Software Version 7: Improvements in Performance and Usability. *Mol Biol Evol*. 2013;30:772-80.

8 Minh BQ, Schmidt HA, Chernomor O, Schrempf D, Woodhams MD, von Haeseler A, et al. IQ-TREE 2: New Models and Efficient Methods for Phylogenetic Inference in the Genomic Era. *Mol Biol Evol*. 2020;37:1530-4.

9 Kalyaanamoorthy S, Minh BQ, Wong TKF, von Haeseler A, Jermiin LS. ModelFinder: fast model selection for accurate phylogenetic estimates. *Nat Methods*. 2017;14:587-9.

10 Hoang DT, Chernomor O, von Haeseler A, Minh BQ, Vinh LS. UFBoot2: Improving the Ultrafast Bootstrap Approximation. *Mol Biol Evol*. 2017;35:518-22.

11 Abramson J, Adler J, Dunger J, Evans R, Green T, Pritzel A, et al. Accurate structure prediction of biomolecular interactions with AlphaFold 3. *Nature*. 2024;630:493-500.

**Supplementary Table**

**Table S1.** A list of VP4 homologues retrieved from IMG/VR v4.
