# Supplementary figures and images for: An archaeal virus capable of hydrolyzing the surface glycan of the host cell
Source: mLife. 2025 Apr 3;4(2):219–22. doi: 10.1002/mlf2.70008 (PMC12042106; doi:10.1002/mlf2.70008)

A

Tree scale: 0.1

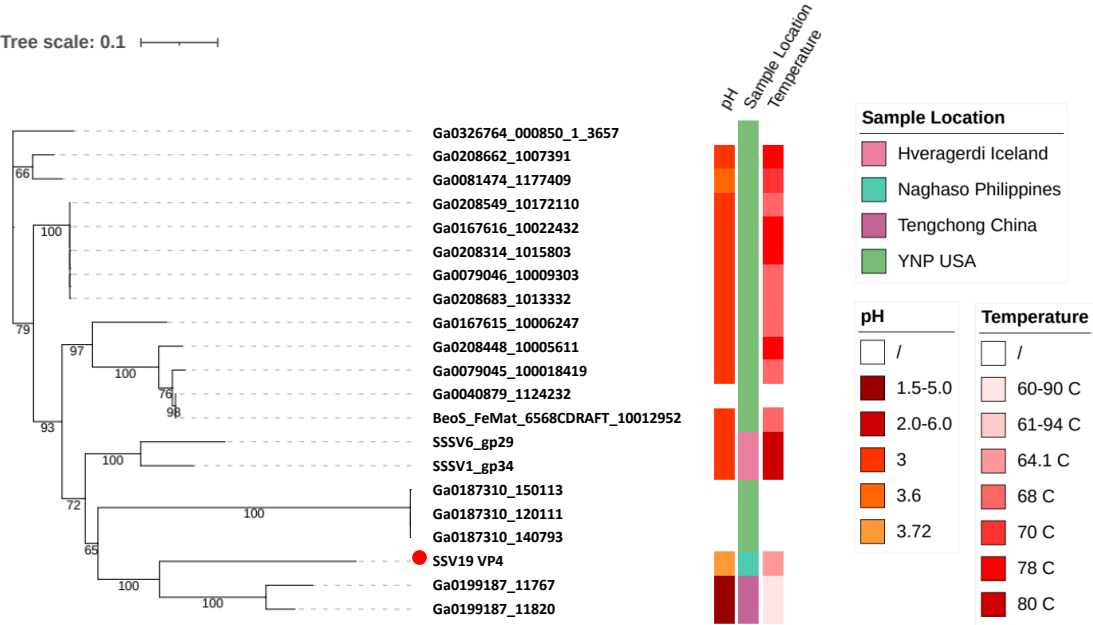

B

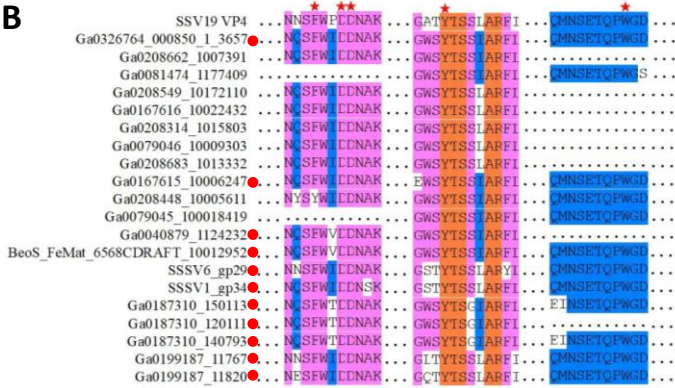

C

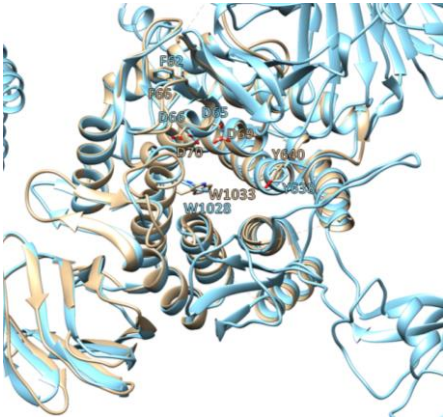

Supplement: Supplementary file 2 — Figure S1. Phylogenetic analysis of VP4. (A) Phylogenetic trees of SSV19 VP4 and its homologues. Proteins homologous to VP4 were collected using PSI‐BLASTP. Details of the analysis are described in the Materials and Methods section. SSV19 VP4 is marked with a red dot. The sampling location, pH, and temperature of VP4 and its homologues are shown in different colors on the right. (B) Sequence alignment of SSV19 VP4 and its homologues. Five key residues (i.e., Phe66, Asp69, Asp70, Tyr640, and Trp1033) in the VP4 core domain are marked with a star. Eleven homologues that have the same five residues with VP4 are marked with a red dot. (C) Comparison of the structural domains of Ga0187310_140793 (cyan) and SSV19 VP4 (brown). The key residues are shown. [file MLF2-4-219-s001.pdf]
